# A qualitative exploration of the phenomenology of pain in children to inform pain assessment methods

**DOI:** 10.1371/journal.pone.0332570

**Published:** 2025-09-24

**Authors:** Thomas F.E. Drake-Brockman, Megan Dodd, Sarah Cooney, Bojana Stepanovic, David Sommerfield, Vance Locke, Aine Sommerfield, Britta S. von Ungern-Sternberg

**Affiliations:** 1 Division of Emergency Medicine, Anaesthesia and Pain Medicine, Medical School, The University of Western Australia, Perth, Western Australia, Australia; 2 Institute for Paediatric Perioperative Excellence, The University of Western Australia, Perth, Western Australia, Australia; 3 Department of Anaesthesia and Pain Medicine, Perth Children’s Hospital, Perth, Western Australia; 4 Perioperative Medicine Team, Perioperative Care Program, The Kids Research Institute Australia, Perth, Western Australia, Australia; 5 School of Psychology, The University of Western Australia, Perth, Western Australia, Australia; Jouf University, SAUDI ARABIA

## Abstract

**Background:**

Pain is a common experience associated with healthcare for children, who often recall it as the worst part of hospitalisation. Several factors make assessment of pain more challenging in children. Families have previously identified the development of improved tools to assess pain in children as a key priority. We therefore sough to investigate the nature of this experience from the perspective of children and their parents to inform the development of such tools.

**Methods:**

To explore the phenomenology of children’s acute pain to inform improved assessment, we conducted a qualitative study with combined (children aged ≥4 and parents) semi-structured interviews of families with children aged 0–16 years at a tertiary paediatric hospital in Western Australia. The interviews were analysed using the Framework Method.

**Results:**

We recruited 27 families, stratified across <6 years, 6–11 years, and 12 + years child age groups. Children and parents demonstrated a range of insights and a wide diversity of ideas about pain. The key themes were: pain is a familiar part of normal life that serves an important function; children communicate pain with others, both verbally and non-verbally; children relate pain to a range of other sensory experiences, with some common experiences identified such as the colour red representing pain; existing pain scales are difficult for children due to limited experiences of extremes of pain; and pain scales could be improved by using more familiar or attractive content or technology.

**Conclusions:**

Children demonstrated diverse experiences of pain and sensory associations. These findings can inform the development of paediatric pain assessment methodology.

The novelty and adaptability offered by digital platforms may assist in creating individualised and engaging pain assessment tools.

## Introduction

Pain is an unpleasant sensory and emotional experience and is critical for identification of clinical deterioration [1]. Assessment of pain can be challenging in children, as they can struggle to describe their pain or to use the scales developed for adults, with no single gold standard assessment tool available [2]. Each year one in 20 children undergoes surgery and 30 million children in the US present to an emergency department [3–5], most of whom will experience pain [6]. Pain can be dominant in the remembered experience of children, who identify pain as the worst aspect of being in hospital [7].

In our experience, routine ward assessment focusses on pain intensity as a single dimension using existing well validated self-assessment tools for children including the 0-to-10 Number Rating Scale (NRS), pictographs (Wong-Baker Faces Pain Scale), linear scales (Pain-Log) [8]. Other approaches include using physical tokens (Pieces of Hurt) or colours (Colour Analog Scale).

These tools often rely on capacities that younger children lack (e.g., seriation of numbers), and common pictographic tools have abstract diagrams that children may not relate to. Recommendations for pain scales vary by age with strengths and pitfalls which have been reviewed elsewhere [9]. These challenges are greatest in young, non-verbal, and developmentally delayed children [10]. In a Western and English-dominant healthcare setting, this may also impact culturally and linguistically diverse children. Parental-proxy or clinical-proxy scales may be used when child report is unable to be obtained, but have the obvious limitation of not being self-reported.

While commonly used, unidimensional pain intensity assessments may be fundamentally inadequate to capture various physical and emotional experiences [11,12] and may not always provide sufficient information to guide management [13]. Pain assessment should expand beyond measurement of pain intensity, and be tailored to each patient [2]. Consumers have also prioritised improved age-appropriate pain assessment tools [14].

Relatively little has been published about the phenomenology of pain in hospitalised children [15]. Understanding children’s experience of pain may improve communication about and assessment of pain [2]. Improving patient comfort may lead to better pain and functional outcomes, improved healthcare experience, and potentially reduced risk of chronic pain [2,16].

We therefore undertook this qualitative phenomenological study to better understand children’s and parents’ experiences of children’s acute pain, to assist in the development of improved paediatric pain assessment tools, including by informing further consumer engagement.

## Methods

Ethics approval was received from the Child and Adolescent Health Service Human Research Ethics Committee (RGS0000005585), recognised by the University of Western Australia Human Research Ethics Committee (2022/ET000897), and registered prospectively with ANZCTR (ACTRN12622001426774). We have applied the COREQ Checklist to the reporting of this study [17].

Strong consumer input was sought through all phrases of this study to ensure a consumer focus and to increase impact and translation potential. This included conversations with a panel of parents and people with lived experience of childhood perioperative encounters diverse in composition, including culturally and linguistically diverse and First Nations members, close involvement of our Youth Consumer Ambassadors (aged 11–15 years), and a Consumer Research Buddy allocated to the project. All patient-facing materials were reviewed by adult and youth consumers.

### Recruitment

We recruited children aged 0 to 16 years along with their parents. Recruitment occurred on the Surgical Short Stay Unit and wards at Perth Children’s Hospital (Perth, Western Australia) from 18 November to 19 December 2022, with convenience sampling. Children and parents were eligible if the child had undergone surgery, presented to the Emergency Department with acute pain and been admitted, or otherwise admitted to the ward with a condition associated with acute pain (e.g. ulcerative colitis flare). For children 4 or more years old, both children and their parents were interviewed; for children less than 4 years old, only their parents were interviewed. Children and parents were excluded if parents were not able to understand sufficient English to consent, as interpreting services were not available for the study.

Recruitment continued until data saturation, defined as the point where no new themes could be identified as determined by consensus of the research team, with an expected recruitment of 25 families. Recruitment was stratified into three age groups: under 6 years, 6–11 years, and 12–16 years, with at least 6 families in each stratum recruited.

Participants were provided with written study information and written parental consent and child consent or assent as appropriate was sought prior to enrolment.

### Data collection

We recorded demographics for children (age, gender) and parents (age, gender, profession), and details of premorbid pain conditions or exposures. Child healthcare literacy was assessed using a provisionally translated version of the HLS-Child-Q15 [18]. The HLS-Child-Q15 was adapted from the European Health Literacy Survey Questionnaire for adults, validated in German for 9–10 year olds [18,19], and translated and validated in Dutch for 8–11 year olds [20]. Parent healthcare literacy was assessed using the Newest Vital Sign (NVS) [21]. Administering the NVS takes 3 minutes and involves asking 6 questions about a nutrition label, with those scoring above 4 unlikely to have low health literacy [22].

Participants completed a semi-structured interview lasting approximately 30 minutes conducted by a trained interviewer (authors TFED-B, MD, SC, and one research assistant). TFED-B and SC are medical doctors, and MD is a student doctor. All interviewers identify as female, except TFED-B who is non-binary. The interviewers included both experienced and novice qualitative interviews and used meetings and tandem interviews to share insights and ensure consistency. The interviewers had no prior relationship with the participants. Participants were aware that the research was about children’s experience and communication of pain, and that we intended to use these findings to inform pain assessment methods.

Interviews were conducted at the bedside with child and parent present, with questions directed to children first, and then to parents to avoid biasing children’s reports. Other family members of the child and parent interviewed may have been present in the room. Interviews were audio recorded and transcribed verbatim. Participants were not provided with the transcripts prior to analysis. The interview commenced with open questions and prompts regarding pain experiences and views following an interview guide developed through consensus of investigators and consumer stakeholders (S1 File). The questions in the interview guide were selectively used and adapted by the interviewers in a responsive manner to encourage discussion by the participants. The questions available were a mix of more open or more closed, with the more closed questions used to confirm or clarify, or to provide an easy question for children who were ‘warming up’ to the interview. Brief notes regarding the interview were made by the interviewer at the end of the interview. There was no follow-up.

### Data analysis

Demographic information was subject to simple descriptive quantitative statistical analysis.

Interview transcripts were subject to thematic analysis following the Framework Method [23], conducted by three researchers (TFED-B, MD, SC), using NVivo (QSR International, Massachusetts, USA; version 14). We used several strategies to ensure trustworthiness, including analyst triangulation. Prior to the analysis each analyst identified and documented their pre-hoc ideas and expectations for subsequent reference. Each analyst familiarised themselves with the interview transcripts and independently inductively coded the first three transcripts, before meeting as a team to discuss findings and agree on a common set of codes. These codes were then used by each analyst to code the same transcript, with discrepancies discussed and resolved before the bulk of transcripts were coded. There were multiple meetings to review discrepancies and achieve consensus throughout the coding process. During coding, consideration was given to the reliability of reports given by interviewees, particularly children, when more closed questions were asked, or if they appeared to be led by the interviewer or a parent. We made a conscious effort to assign little weight to simple agreement with an idea stated by somebody other than the child. The coded transcripts were charted onto a Framework Matrix, with key participant quotes also identified.

During charting, key ideas raised were identified and summaries were written by the analysts. The summaries also relied on a broad understanding of all transcripts to draw insights from the entire qualitative dataset. These summaries were the primary source of the synthesis and themes, which were reviewed by all authors. There was no participant input into the qualitative analysis process, however the Consumer Research Buddy assigned to the project was consulted.

A secondary post-hoc quantitative analysis was conducted to calculate counts of participants who raised specific ideas within codes. These counts were determined by clustering the ideas within each code charted onto the Framework Matrix. This was done using a novel human-augmented machine learning process: ideas were embedded into a highly dimensional vector space using the all-MiniLM-L6-v2 model for sentences [24], then clustered using the sklearn package [25]. These were and then reviewed and adjusted by the first author.

Further numeric analysis (e.g., calculation of proportions) was not done as the counts presented are not intended to estimate the population, but rather to illustrate the volume of reports. This secondary analysis did not influence the key themes identified.

## Results

We approached 36 potential participants, with 27 (75%) recruited. One interview abandoned due to the child’s clinical condition was excluded from analysis. Two interviews included two parents; the remainder included one parent. Recruitment stopped after 26 complete interviews as data saturation was achieved. Demographics are presented in Table 1.

**Table 1:** Demographics of children and parents interviewed.

Child Demographics	
n	26
Age	8.5 (1–16)
Gender	13 Female, 13 Male
HLS-Child-Q15 Score	3.37 (1.73-4.00)
Pain Exposures	
Abdominal pain	8
Trauma	5
Orthopaedic surgery	4
Pelvic surgery	2
Severe pneumonia	2
Colorectal surgery	1
Ear pain	1
ENT surgery complication	1
Facial pain	1
Neurosurgery	1
**Parent Demographics**	
n	28
Age	37 (21-51)
Gender	24 Female, 4 Male
Newest Vital Sign Score†	5.5 (1.0-6.0)

Values are median (range) or count.

†Only completed by one parent per interview, primary caregiver if present

An expanded exploration of the ideas identified by children and parents is contained in S2 File.

### Pain as a concept and experience

Children described various kinds of pain and understood that it had a purpose. They understood pain as indicating tissue injury (12) or a signal to seek help (7). Some viewed pain as a universal human experience and a normal part of life (4). Emotional or mental pain was recognised (4). Children generally described pain as a distinct sensation.

“It’s a bodily function, to allow you to know what’s going on- like If you need something done, if you have broken something or if you have hurt your body.” – QU06, Child

Parents saw pain as a sign something is wrong (13), a normal part of life (7), a clue of injury (7), a trigger to seek help (4), or as a physiological function (1). Many saw both emotional and physical pain in their children (7), noting that pain expression could vary in line with a child’s disposition.

### Communicating pain

Children found it easy to recognise when they experienced pain (3), to tell somebody about it (3), or ask for help (3). Generally, they reported pain to parents and family (5). Many children found it easy to describe (6) or communicate pain (5), though a few (2) were unsure or found this difficult. Some mentioned stoicism (1), unfamiliar painful experiences (1), or coping (1) as barriers to others understanding their pain.

“Ah well, when I talk to the doctors and stuff they find strategies to understand me. If they don’t understand me they’ll find a way. But usually every time I talk to a doctor and say… tell them how I fell and stuff they usually get on top of it. Because that’s their job. They’ve got to do that.” – QU02, Child

Parents noted children communicated pain verbally (4) and were confident in their children’s ability to express this (2). Several parents (5) identified intuition or ‘knowing their child’ as key to recognising pain. They noted that factors including personality (4), experience or resilience (3), neurodiversity (1), speech development (1), communication skills (3), or medication (1) could affect their children’s report of pain.

### Verbal communication

Most children reported that they were comfortable communicating their pain verbally (9), using a variety of words and nouns to describe the feeling. Words used specifically to identify pain by children included: “ouch”, “sore”, “pain”, and “hurt”.

“Yeah I would tell her where the area was. And I would tell her that it feels… Like how the pain felt out of 10. I would tell her out of ten. And I would tell her… or ummm… like how it felt like if it was sharp pain or it was just tender or if it was like tingling like I would use describing words to kind of… show her… yeah.” – QU01, Child

Many parents reported that their child could and did report pain verbally (5), although some reported this was variable (2) or particularly so for severe pain.

### Non-verbal communication

Some children acknowledged using non-verbal communication and reported crying (5), screaming (3), and pointing to the affected body part (3).

“But a 10 you would be, like, extreme, like screaming, yelling and like crying out in pain, you know.” – QU06, Child

All parents reported non-verbal communication in detecting their child’s pain including body language or facial expression (8), screaming, crying, or moaning (3), or through emotional state (6) or mood (3). Parents noted this particularly for younger children (3). All parents noted there was a level of ‘just knowing’ when their child was in pain and using their intuition.

### Who pain is communicated to

Children reported that they communicate their pain to familiar people like a parent (14), sibling or family member (4), friend (5), or teacher (3). Most were comfortable communicating pain with healthcare workers, including doctors (6) and nurses (5), and felt understood. Many children reported that healthcare workers, as experts, would understand their pain (7).

“Because you know when you are feeling bad and you’ve just got to tell your mum and dad that you are feeling bad.” – QU22, Child

Parents reported that their children usually report pain to them (parents) (14), other family members (4), teachers, school staff, or authority Figures (7). Most parents expressed that healthcare workers understood children’s pain (13).

### Describing pain

Children noted specific ways they describe pain and the understandings underpinning these descriptions. They noted varying levels of pain intensity (6) and adjusted their expressions accordingly. Some recognized different types of pain (7), including emotional and physical pain, and related pain and sickness (4). Some related pain to anxiety or fear (7). Descriptors used included pressure, bad, tense, awkward, sensitive, heaviness, ringing, pounding, tender, agony, aching, throbbing, and stabbing.

Parents often reported similar words as their children, including “hurts” (12), “ouchie” (7), and “sore” (3). Some parents noted their children will describe the character of their pain – for example burning or stinging (3), shooting or stabbing (3), discomfort (3), or pounding or throbbing (2).

### Associations with pain

Children reported a strong consensus that the colour of pain is red or blood-coloured (16), or black (5). A variety of shapes were used to describe pain (13), usually those with sharp or spiky features. Children (5) identified unpleasant smells that they associated with pain, and that pain would sound like screaming (4), or other harsh sounds. Some children described that pain would feel sharp (4) or hot (2).

“Ahhh well if it was broken it would be sharp maybe. And if it was like a sting it would be smooth and… maybe a bit bumpy.” – QU12, Child

A few parents reported what pain might look like. Parents identified that red (5) and darker colours (2) represented pain. Few parents reported on olfactory, auditory, or tactile associations with pain.

### Using pain scales and novel ideas

Many children demonstrated understanding of pain scales through examples and expressed satisfaction or familiarity (6). Some children reported barriers (3), such as distinguishing between numbers and having limited personal experiences of pain to anchor scales against.

“Because they can understand it more. Like in the 0 and 10 thing, I think that would be more a big kid thing. Because they know what they are talking about. But a little kid can just move the scale.” – QU12, Child

When asked for ideas for novel pain scales, many children suggested using colours (9) such as a traffic light system (red, orange, green). Some also suggested pictures instead of numbers (4). A few children suggested that technology could enable age-adjusted scales (1), options for children with disabilities (1), and allow children to self-update pain scores (1).

Some parents reported that existing pain scales were useful, and that their children understood and used them appropriately (3). However, more parents felt the scales were not helpful (7), especially for younger children. Suggestions included scales being more engaging or easier for children to understand. Suggestions included picture scales (7), using colours (9), familiar characters, or more interactivity.

Parents suggested technology could aid pain assessment and engagement, particularly for children with special needs (3), though concerns about usability (1) and financial barriers (1) were raised.

“Yeah maybe like an app where that can touch the screen where they can touch the colour, where it hurts. Especially for those who are non-verbal. With special needs who can’t express, or have those higher pain thresholds, but its, yeah. I don’t know. But an app would be good.” – QU11, Parent

## Discussion

This qualitative study demonstrated a range of insights into the experiences and communication of pain. While in some areas children and parents independently offered similar ideas, more striking was the diversity of ideas offered. This study addresses our fourth-ranked consumer research priority “Developing more effective and age-appropriate tools to address children’s anxiety, level of pain, and understanding of the hospital process” which is also linked to the second-highest consumer priority “Reducing fear and anxiety in children throughout the hospital experience” identified in an Australia-wide consumer research priority-setting project [14]. We believe this study is a significant contribution to the in-depth qualitative phenomenological exploration of children’s pain in English-speaking children, and the most in-depth exploration of sensory association.

The key themes were: pain is a familiar part of normal life that serves an important function, children communicate pain with others – both verbally and non-verbally – and usually feel confident to do so with people they are familiar with, children relate pain to a range of other sensory experience with some commonality in these experiences, existing pain scales are difficult for children to use due to limited experience of extremes of pain, and improved pain scales could use more familiar or attractive associations or the use of technology.

### Pain as a familiar part of normal life

Children identify pain as a familiar concept, but one that is often challenging to describe. Children know that pain is useful and protective, as has been previously identified [15]. In many interviews it was clear, if not explicitly stated, that children were confused why we were asking what pain *was*, as if pain was considered by these children to be axiomatic.

Some children volunteered that pain could be emotional, in the absence of physical insult. It has been previously been identified that children equate pain with fear, anxiety, and emotional insult [26,27]. We ask if these emotional pain experiences are what we wish, or expect, to measure when asking about “pain”, and how tools can identify or differentiate kinds of pain.

Parents are familiar with seeing pain in their children. Some parents struggled with what pain was, but many more explained it either in terms of a protective instinct, body function, or help seeking. Parents were more likely to think about pain in terms of physical injury, perhaps because they have a greater nomenclature for other distressing emotional experiences that children might liken to pain. We have previously found a good level of agreement between child and parent ratings of pain [28].

### Communicating pain with others

Children and parents both report that children verbalise their pain, and a variety of non-verbal aspects of communication about pain. Children usually and preferentially report pain to their parents, suggesting that creating a safe and familiar environment may assist children with reporting their pain. Barriers were identified to non-parents understanding children’s pain, including for healthcare workers, although some children and parents felt that paediatric healthcare staff may have particular insight in this area.

Often children reported that their verbal report was to directly identify that they had pain, perhaps using one of range for words *for* pain such as “hurt”, “ouch”, or “owie”. Children are known to use a range of vocabulary and non-word vocalisations, which varies with age and development [29].

Parents noted a wider range of non-verbal communication including emotional state, mood, behaviour, and activity -– seeking to detect signs of suffering below the child’s threshold to report. Parent’s intuition (implicit knowledge) of their child was identified as a useful surrogate for self-reporting of pain, suggesting that pain assessment tools could be ‘calibrated’ according to parental knowledge of their child.

### Relating pain to other sensory experience

Children showed a high level of imaginative thinking in variously relating their experience of pain to other sensory experience discussed, e.g., colour, shape, movement, and pain imagery., A few common themes stood out: pain is red or black, and to a lesser extent it is sharp or spiky in appearance. Red and then black have previously been commonly identified as colours for pain [30], however adding shape and texture to tools may also be helpful. The colour red was also prominent for parents. Children also suggested other unpleasant or jarring sensory experiences associated with pain, such as fingernails on chalkboard. Finnish children have described pain as “the most disgusting experience” and being “yucky” [15,31].

### Using or improving pain scales for children

Asked about a better scale, children offered roughly two sorts of ideas – abstract and tangible. The abstract associations were typically colour, but also included using weather or emojis. The tangible examples generally related to injury (e.g., a picture of a limb in a cast). Parents make some similar comments regarding pictures or colours, and also comment on making scales more attractive to children by using familiar media, or being more interactive. Children prefer scales with faces in them over more abstract scales (e.g., CAS), which may be due to familiarity [32–34], and prefer digital delivery of pain scales [35]. Digital delivery can offer efficiencies for staff such as immediate recording of results [36]. Both children and parents commented on benefits of technology (e.g., an iPad) for presenting pain scales.

### Limitations

This study has several limitations that should be noted, including that only English-speaking families were included. We used a single-interview methodology without follow-up, and did not review transcripts with participants to clarify their intended meanings. We used a combined interview format with both child and parent for children older than 4 years old, and only interviewed parents of children under 4 years of age. We studied an inpatient population, and hospitalised children have been found to describe pain differently from non-hospitalised children [30].

We found the use of consumer engagement to be useful and would recommend that similar studies or replications undertake this process. The combined interview format worked well, and we would not change this if repeating the study. Recruiting a broader population of children, such as those presenting with pain in primary care settings or living with chronic pain, would assist in determining how broadly these findings can be applied.

### Future work

This study has implications for further research, with a wide range of ideas that could be explored further and validated. This could include mixed-methods or quantitative research to assess associations between suggested pain scale elements (e.g., correlating colours with 0–10 pain intensity axis location) in a larger cohort. The use of other sensory descriptors and willingness to use digital platforms could allow more complete and multidimensional assessment of the different types of pain described. The novel ideas expressed can also inform discussions and co-design methods with consumers as conversation starters, or to allow additional ideas to be incorporated in these processes.

## Conclusions

In this qualitative phenomenological study, we explored experiences, descriptions, and communication of pain by children. The diversity of these ideas demonstrates the individuality of the pain experience, and the impact of children’s prior exposures. Parents also provided a range of responses, with strong common themes emerging in the familiarity of pain as a common unpleasant experience, and children’s willingness to ascribe sensory association to their pain experience.

This diversity of children’s pain experience and mixed views on existing pain scales suggests that scale selection needs to be individualised. The range of descriptions and associations offered by children should be a target of interest of healthcare professionals at the bedside, as enquiry with a child or parent about how they perceive and communication pain could assist assessment and monitoring for that child. Digital platforms may be able to assist with providing both individualised assessment tools, as well as a more engaging experience.

## Supporting information

S1 File Interview guide. The interview guide used when conducting interviews with study participants. (DOCX)

S2 File Exploration of themes. A detailed exploration of the ideas and themes contained in the interview transcripts with accompanying quotations. (DOCX)
