# Supplementary material for: A qualitative exploration of the phenomenology of pain in children to inform pain assessment methods
Source: PLoS One. 2025 Sep 24;20(9):e0332570. doi: 10.1371/journal.pone.0332570 (PMC12459796; doi:10.1371/journal.pone.0332570)
Supplement: S1 File — (DOCX) [file pone.0332570.s001.docx]

Interview guide

# Note to Interviewer

As this is a semi-structured interview, it is expected for the participant to deviate from the question. In the event this happens, remember to revisit, prompt, and reword the question. Feel free to explore specific themes or clarify points that are raised by the participants.

While some questions are intended to be directed to either the child or their parent, it is anticipated that they will both have something to contribute for many questions, which is to be encouraged.

# To start the interview

I’m going to ask you some questions about how you feel about pain. We want to know your thoughts and are really interested to hear what you have to say. There are no right or wrong answers, and you do not need to answer any questions you don’t feel comfortable about.

Would you (addressing both parent and child) prefer to talk to us together or separately?

# Children’s perceptions of their ability to understand and communicate their experience of pain

Questions for children about understanding pain:

- What do you think pain is? How easy or hard is it to know?
- Why do you think pain happens? How easy or hard is it to know?
- What do you think pain means to you?

Questions for children about communicating pain:

- How do you tell other people about pain? How easy or hard is it to do?
- Who do you tell about pain? Do they understand?
- Do other people understand what you say about pain?
- Do your parents understand? Do doctors understand? Do nurses understand?
- Do you have any thoughts on why that is?

# Parental perceptions of their children’s ability to understand and communicate their experience of pain

Questions for parents about children’s understanding of pain:

- What do (your) kids think pain is? Is it different from what you think?
- Why do (your) kids think pain happens? Is it different from what you think?
- What do (your) kids think pain means? Is it different from what you think?

Questions for parents about communicating pain:

- How do (your) kids tell other people about pain? How easy or hard is it for kids to do?
- Who do (your) kids tell about pain? Do you think they understand?
- Do other people understand what (your) kids say about pain?
- Do parents understand? Do doctors understand? Do nurses understand?
- Do you have any thoughts on why that is?

Questions for parents about recognising pain:

- Do other people recognise when kids have pain?
- Do parents recognise it? Do doctors recognise it? Do nurses recognise it?
- Why is that?

# Children’s use of different words/concepts to identify the experience of pain

Questions for children about words that relate to pain:

- What other words do you use/know for pain? (e.g. “hurt”, “sore”, “ouch”)
- Are there other words that are not quite the same but a bit like pain?
- What words do you use when you are worried about pain?
- What words mean the opposite of pain? (or opposite of other words used above)
- What words do you use when you are not worried about pain?

Questions for parents about children’s words that relate to pain:

- What other words do (your) kids use/know for pain? (e.g. “hurt”, “sore”, “ouch”)
- Are there other words (your) kids use that are not quite the same but a bit like pain?
- What words do (your) kids use when they are worried about pain?
- What words do (your) kids use to mean the opposite of pain? (or other words used above)
- What words do (your) kids use when they are not worried about pain?

# Associations between experiences of pain and other sensory experiences

Questions for children about experiences of pain:

- What is pain like? What kinds of feeling is pain? What does it feel like?
- What makes pain different from other bad/unpleasant feelings?
- How would you explain pain to somebody/an alien who didn’t know what it was?

Questions for children about sensory associations with pain:

- What does pain look like if it was a thing?
- What colour is it? What shape is it? How big or small is it?
- Does it move? Does it blink or flash?
- What does pain sounds like if it was a sound?
- Is it loud or soft? Is it squeaky or rumbly? Is it slow or fast?
- What does pain smell like?
- Does it smell like anything else you know?
- What does pain feel like if you could touch it?
- Is it soft or hard? What texture does it have? What temperature is it?
- What would happen if you poke it?
- What does pain taste like?
- Does it taste like anything else you know?

Questions for children about sensory associations with contrasting experiences (can be described as "no pain”, “the opposite of pain”, “feeling comfortable”, or whichever way is best understood):

- What does *no* pain look like if it was a thing?
- What colour is it? What shape is it? How big or small is it?
- Does it move? Does it blink or flash?
- What does *no* pain sounds like if it was a sound?
- Is it loud or soft? Is it squeaky or rumbly? Is it slow or fast?
- What does *no* pain smell like?
- Does it smell like anything else you know?
- What does *no* pain feel like if you could touch it?
- Is it soft or hard? What texture does it have? What temperature is it?
- What would happen if you poke it?
- What does *no* pain taste like?
- Does it taste like anything else you know?

If the child can identify different sensory experiences for “pain” and “no pain” (or another contrasting experience) then also ask what the space between those experiences is like. For example, if “pain” is orange and “no pain” is purple, what is between those two?

# Ideas about communicate their experience of pain using scales

Briefly explain what a pain scale is and how it works using examples (NRS, WBFS).

Questions for children about communicating pain using scales:

- If you were designing a pain scale for kids, how would you design it?
- Would it work for younger kids? Would it work for older kids?
- Would it work for kids who can’t talk or use words?
- Would it work for kids who can’t count or use numbers?
- Do you have any other ideas?

Enquire about colours, shapes, forms, noises, motion, and any other features.

Questions for parents about communicating pain using scales:

- If you were designing a pain scale for (your) kids, how would you design it?
- Would it work for younger kids? Would it work for older kids?
- Would it work for kids who can’t talk or use words?
- Would it work for kids who can’t count or use numbers?
- Do you have any other ideas?

Enquire about colours, shapes, forms, noises, motion, and any other features.

# To conclude the interview

Thank you for your time and for answering those questions. Do you have anything else you would like to say?
