# Supplementary material for: A qualitative exploration of the phenomenology of pain in children to inform pain assessment methods
Source: PLoS One. 2025 Sep 24;20(9):e0332570. doi: 10.1371/journal.pone.0332570 (PMC12459796; doi:10.1371/journal.pone.0332570)
Supplement: S2 File — (DOCX) [file pone.0332570.s002.docx]

Exploration of themes

# Pain as a concept and experience

When children spoke about pain as a concept, they described pain as being of various kinds, and of having a purpose. Children were aware that pain was an indicator of tissue injury (12), or as an indicator or warning signal that told them that something was wrong and that they needed help (7). Some children expressed that pain is a universal human experience and a normal part of life (4) and something that, while unpleasant, we all experience. Some children indicated that pain limited their activities (4).

“It’s a bodily function, to allow you to know what’s going on- like If you need something done, if you have broken something or if you have hurt your body.” – QU06, Child

“Uhm I would say it’s part of life, you need to get through it. Its, you going to do it about a million more times in your life.” – QU08, Child

“I feel like I need it sometimes. Just so I know if something is wrong. And if I need help.” – QU12, Child

“Ahh well the nerve pain is just really sensitive. Nerve pain a little bit different it can happen either when a bone is removed or um can be caused from an accident as well, from having like a brain injury or anything. Um but really it…nerve pain is like different from other pain as well. And that physical pain from when you get hit or something, nerve pain is a lot different.” - QU02, Child

There was an awareness of pain being not only a consequence of physical affliction, as children also recognised emotional or mental pain (4). Some children described this as a separate pain.

“I think pain is, two types of pains, one when you feel depressed, and another type of pain when you feel hurt, so when you’ve fell down and scratched your knee or something” – QU29, Child

Children generally described pain as an abstract or discrete feeling or sensation, rather than associating pain with specific symptoms or signs. Some children did associate pain with low energy and difficulty moving (3), tingling (1), crying (1), and being pale (1).

“Umm, probably less energy. Less energy than you usually do when you are in pain. Because when you are in pain you don’t really want to start moving around all happy and joyful and stuff.” – QU02, Child

When speaking to parents, a single theme emerged repeatedly: pain is an indicator that something is wrong (13). For many parents, pain was viewed as a normal part of life (7), as a clue or sign of injury (7) to be further explored with their children, a trigger to seek help (4), or specifically as a physiological function (1). They thought, from a child’s perspective, that pain was a trigger to seek help (3). Many parents also flagged that they saw both emotional and physical pain in their children (7).

“I mean he’s a normal kid. He’s a human. We get pains all the time. I can just tell like if he’s injured himself quite badly it’s quite normal to have pain.” – QU02, Parent

“Because there is something wrong. Something is bothering them. Or something is not right.” – QU21, Parent

“I think it is their way of telling us if they’re sick, so we know.” – QU13, Parent

Parents described a large range of signs that suggested their child was in pain. This included physiological observations such as fever or raised heart rate (8), subjective observations of paleness or reduced appetite, and other clues in the child’s behaviour (7) or activities (6). Parents also express that pain could be experienced differently according to the child’s baseline disposition, manner, personality, and idiosyncrasies. Often parents identified that they know that something was wrong due to factors including changes from their child’s normal behaviour, body posture, facial expression, personality, or mood.

# Communicating pain

Most children who discussed communication reported that it was easy to know when they were experiencing pain (3), or that it was easy to tell somebody about it (3) or ask for help (3). In general, children reported that they tell their parents and families about pain (5), as well as other people they feel familiar with. Some will tell healthcare workers about their pain or seek medical care (4).

“Ah well I normally just go up to them and say, ‘ah I hurt myself’.” – QU33, Child

“Uhm, you sometimes ask ‘Can I have like Panadol [Paracetamol], or medicine?’ Uhm or you tell someone that you are feeling sore, and you tell them where you are feeling sore so that they know where its sore.” – QU05, Child

There were a range of comments from children around efficacy of communication. Many stated that pain was easy to describe (6) or easy to communicate (5), although a few (2) were unsure or found this difficult. A few children identified that closed-loop communication (including clarifying statements) could assist with confirming that healthcare workers understand (2) – such as when doctors offer additional words to establish that they have a common understanding – or that healthcare worker expertise was helpful (1).

“They start like listing things, similar things, then if it is similar then they can get an idea of what I am dealing with.” – QU11, Child

"Ah well, when I talk to the doctors and stuff they find strategies to understand me. If they don’t understand me they’ll find a way. But usually every time I talk to a doctor and say… tell them how I fell and stuff they usually get on top of it. Because that’s their job. They’ve got to do that." – QU02, Child

A few children raised that being stoic (1), having unfamiliar painful experiences (1), or coping with their pain in an unexpected manner (1) were barriers to how others understood their pain.

“I was trying to act tough, but I was a scared to feel the pain as well. So they might be a bit scared of how strong it might be or so they just try to fight it and that’s usually not the way to do it.” _ QU02, Child

Parents had a wide range of comments on communication of pain by children. Parents reported that their children communicated their pain verbally (4) and conveyed confidence in their children’s ability to express themselves, but also that the ability or propensity to do this was variable (2). A few parents identified that they were more able to discern their child’s pain than others, including healthcare staff.

“I think he understands pain, but I don’t think he understands how to express that he is in pain. So the only way he feels therefore that he can express pain is by crying, screaming, kicking and being super grumpy.” – QU19, Parent

“I think sometimes it can be hard to communicate it in terms of the level of the pain. Yeah so… they can experience pain and tell you but it’s level… ahh for instance my three-year-old recently broke his arm. For him to pinpoint where on the arm exactly, where the exact pain was a hard thing. He kept touching everywhere. So, defining the pain sometimes is hard depending on the age group.” – QU22, Parent

“But then there were other times where I think [staff] underestimated the amount of pain he was in, or perhaps they confused it with ah behavioural issues. There have been a couple of times were that has happened.” – QU19, Parent

“I think that people who know her well would [know] but people that don’t know her wouldn’t realise.” – QU35, Parent

Regarding efficacy of communication, several parents (5) identified that their parental intuition, gut feeling, and ‘knowing their child’, were particularly important in recognising their child’s pain. A few parents noted that the effectiveness of a child’s report could be impacted by factors including personality (4), experience or resilience (3), neurodiversity (1), speech development or delay (1), as well as development of verbal and communication skills (3) or could be masked by medication (1). Some parents expressed concern over not being able to recognise their child’s pain or feeling a sense of guilt when they had not noticed the severity of their child’s pain.

# Verbal communication

Most children reported that they were comfortable communicating their pain verbally (9), using a variety of words and nouns to describe the feeling. Children associated expressing their pain with asking for help (5), specifically, seeking support or medication from a parent. For some children, requesting medicine was how they communicated their pain (4). Words used specifically to identify pain by children included: ouch, sore, pain, and hurt.

"Yeah I would tell her where the area was. And I would tell her that it feels… Like how the pain felt out of 10. I would tell her out of ten. And I would tell her… or ummm… like how it felt like if it was sharp pain or it was just tender or if it was like tingling like I would use describing words to kind of… show her… yeah." – QU01, Child

Many parents reported that their child could and did report their pain verbally (5), although some reported that their child did so variably (2) or particularly so when their pain was severe.

“He does. He cries, he says ‘It hurts’ and he show us where it hurts.” – QU06, Parent

# Non-verbal communication

Some children also acknowledged the non-verbal side to their communication and reported crying (5), screaming (3), and pointing to the affected body part (3) as their predominant forms of non-verbal communication.

“But a 10 you would be, like, extreme, like screaming, yelling and like crying out in pain, you know.” – QU06, Child

“I was grabbing my stomach, trying to indicate that it hurt.” – QU11, Child

All parents reported an element of non-verbal communication in detecting their child’s pain including aspects of body language or facial expression (8), screaming, crying, or moaning (3), or through emotional state (6) or mood (3). Parents noted that this was more so with children who were younger (3), when they might rely more on crying and screaming to understand their child’s pain. For parents with older and more verbally communicative children, parents noted that they differentiated high levels of pain based on their child’s body language. Regarding this more severe pain, parents reported noticing agitation, restlessness, cuddliness, and grumpiness. All parents noted there was a level of ‘just knowing’ when their child was in pain and using their intuition.

“She will kinda groan, like groan asleep, even it will wake her up out of here sleep. Which is hard to tell if you don’t know the child and their sleep pattern normally. She will be coming quite irritable, quite demanding. Like she’s happy for me to sit away from her and then in the middle of the night she will be like oh I need your next to me.” – QU18, Parent

“I see how her facial expressions and, in the place, that she’s telling me that is hurting her. She is bending towards that place, and she can’t stand straight, and she can’t walk properly, she needs a wheelchair.” – QU29, Parent

# Who pain is communicated to

Children reported that they were happy to communicate their pain with a range of people in their lives. Of children who commented, most () preferred to tell somebody that they are familiar with – a parent (14), sibling or another family member (4), a friend (5), or a school teacher (3). Usually, children reported that they were also happy to communicate their pain with healthcare workers including doctors (6) and nurses (5) and that their pain is understood. However, some children identify that their parents (particularly their mothers) are more able to understand or more able to provide comfort than healthcare workers, who they might find unfamiliar or intimidating (2). Many children reported that healthcare workers are experts or have training that they expect will enable them to understand pain experiences (7).

“I would tell my mum or my dad, and I would say that its sore and I would say where it’s sore” – QU35, Child

“Because you know when you are feeling bad and you’ve just got to tell your mum and dad that you are feeling bad.” – QU22, Child

Parents reported that their children most commonly report pain to them (parents) (14), but also to a range of other familiar people including other family members (commonly grandparents) (4), teachers and other school staff (e.g. school nurse), and other authority figures in the child’s life (e.g. sports coach) (7). Some parents suggested that their children will report pain to their friends (2), although it is less clear if this is outcome-directed (e.g. to seek help). A few parents raised that their child sought to report pain to healthcare staff (2), although it seemed that many children are content to pass this information on via their parents.

Most parents felt that healthcare staff did understand children’s pain (13), although some parents felt that it was a different kind of understanding from their own. Some parents identified nurses as having a different understanding to doctors also, perhaps related to greater or more regular bedside contact (1). A few parents also reported on how they thought clinicians gleaned insight into a child’s pain including through sources of information (4) such as test results, vital signs, close clinical assessment, or through observing response to medication (2). Some parents reported that clinicians who specialise in the care of children have additional insight into paediatric pain (2), with a few parents suggesting that this specialist insight may even be more attuned than parents in some circumstances. It was noted that parents may be able to assist healthcare staff to understand their child’s pain (2). Other than noting the value of parental insight, several parents observed that the person who receives a pain report, may themselves have various preconceptions or biases that effect how the communication about pain is received.

# Describing pain

Children commented on both specific ways that they describe pain and the understandings that underpin these descriptions. Children noted that there are varying degrees of pain intensity (6) and that they adjust their expression of pain accordingly. Some children also reported recognising different types of pain (7), including emotional pain and physical pain, and the relationship between pain and sickness (4). Some children related talking about their pain to feeling anxiety or fear (7). A range of descriptors were offered including: pressure, bad, tense, awkward, sensitive, heaviness, ringing, pounding, tender, agony, aching, throbbing, and stabbing. Some children admitted they were unsure how they would describe pain (2).

When children were probed about feelings that were like pain but not the same, they mostly reported this as being some form of struggle (5), or in relation to anxiety (4) or sickness (3). Many children struggled to describe the feeling of not being in pain. Those who were able to, described it in terms of being neutral or free from adversity (9), or being positive and happy (7), calm (3), or of strength and vitality (2).

Parents often reported similar words as their children did for pain. Parents commonly reported that children used the words “hurts” (12), “ouchie” (7), and “sore” (3) to describe when they are in pain. Some parents mentioned that their children were frustrated or angry about being in pain (3), noting that they may say the pain is “too much” or “not fair”. Some parents reported that their children will use words that describe the character of their pain - for example burning or stinging (3), shooting or stabbing (3), discomfort (3), or pounding or throbbing (2).

“Usually it’s an ache, aching, feeling you know, depends on what part of their body as well, so like they’ll say if it’s a headache they’ve got a sore head or sore eyes, things like that. Heavy. You’ve told me, when you’ve had tingling in your fingers, or a heaviness when pushed.” – QU11, Parent

Parents found the question of what words would their child use for the opposite of pain to be challenging. The predominant words that parents reported positive words such as “happy” or “good” (13), words expressing an absence of adversity like “better” or “fine” (9), being “comfortable” or “relaxed” (7). Parents also noted that their children might simply be happy, chatty, or smiling (5). Parents usually reported understanding no pain as being the baseline experience of their children when they are their normal selves. In relation to the alternative experiences to pain, parents tended to comment on the notion of their child being sick (3), scared (3), or anxious (3). Parents also found it difficult to answer about how their children described feelings that were like pain but not the same.

# Associations with pain

When asked what pain would look like children give a wide variety of responses. Children reported a strong consensus that the colour of pain is red or blood-coloured (16) or black (5). A variety of shapes were used to describe pain (13) and tended to be those that have sharp or spiky features. The opposite of pain was more likely to be described in terms of a familiar pleasant experience (e.g. at the beach) or with imagery or symbols (heart, smiley face, rainbow, etc). There were a few children who suggested green and/or yellow as the opposite of pain, but there was no strong consensus.

“Imagine a little ball and its calm. And then all of a sudden it just gets crunched up. So, like a stress ball. So, like putting all your pain and stress into a little ball and squeezing it. So, it’s kind of like a cramp just going all together.” – QU02, Child

“Ahhh well if it was broken it would be sharp maybe. And if it was like a sting it would be smooth and… maybe a bit bumpy.” – QU12, Child

“So, I think maybe pain, would look like [a] three headed monster. … with three stiches, with all these different stitches, with each pain that it’s felt before and then it’s all black, everything in its mouth is red. That’s what I think it would look like. If I saw that in real life I would be petrified.” – QU29, Child

Children (5) identified several unpleasant smells that they associated with pain, with a few children stating that pain smelt like blood or generally stinky/rotten. A few children identified that no pain would smell pleasant (5), electing nice foods such as strawberries or apple pie. Children identified that pain would sound like screaming (4), or similar harsh sounds such as banging (1) or the sound of nails being drawn across a chalkboard (1). A few children (3) identified that no pain might sound like pleasant sounds of rain, seaside, children playing, or giggling. Some children identified that pain would have an unpleasant taste (5), with examples including charcoal, sprouts, or a metallic taste. Some children described what pain might feel like if touched, as sharp (4) or hot (2), and several identified that no pain would be soft or gentle (4).

A few parents reported what pain or no pain might look like. Parents identified that red (5) and darker colours (2) represented pain, whereas light, pastel, or bright colours represented less pain (2). Some parents noted that sharp or irregular shapes could represent pain. A few mentioned other shapes, faces, or characters, without any consensus. Few parents reported on olfactory, auditory, or tactile associations with pain or no pain, although those who did mentioned that harsh drums, screaming sounds, or sharp and spikey tactile experiences could be associated with pain.

# Using pain scales and novel ideas

When children were asked about existing pain scales they had used (commonly the 0-10 verbal NRS), many children reported or demonstrated understanding of how a pain scale worked through examples. Children expressed satisfaction or familiarity with the use of the scale (6) in assessing their pain. Children also reported barriers to using the current pain scale (3), identifying difficulty understanding how to distinguish between all the numbers and the risk that the pain scale was limited by having few personal experiences of pain to anchor the scale against.

“Because they can understand it more. Like in the 0 and 10 thing, I think that would be more a big kid thing. Because they know what they are talking about. But a little kid can just move the scale.” – QU12, Child

“So, it’s like if it’s over a 5 it’s kind of more emergency like if I would want to have them come. But if it’s under 5 it’s like not as serious. Like I was still in pain, but like it’s not as big.” – QU01, Child

“Like we say 10 is the most excruciating pain you ever felt – she was in more pain the other day and she’s still saying 10 now like it’s quite clear that it’s not as bad.” – QU33, Parent

When children were asked for their ideas for novel pain scales a range of suggestions were made. Most commonly, children focused on the use of colours (9) and a traffic light system (red for worse pain, orange for medium intensity, green for less pain). Other colours suggested by children were dark colours or black for pain and light pastel greens and blues for no pain. Children suggested using pictures instead of numbers (4), however, each child's individuality prevailed as to what these pictures should be. Some children suggested using pictures of actual injuries to compare the pain to, and others suggested weather patterns, and emojis to relate the emotion the pain incited.

A few children commented when asked about the use of technology in novel pain scales and raised interesting points. It was suggested that pain scales could be streamed or adjusted based on a child’s age (1), that it could be a helpful way to incorporate different sensory options for children with disabilities (1), and could allow a child to regularly update their pain score themselves (1) – i.e. alerting as soon as there was a change.

“Now I’m thinking about what matters to me. Maybe you can create an app, if they don’t want to write, if they can’t write, they only press buttons, so you could do that? Or maybe you could just like – they can do the have your say thing on the TV? If they feel scared to talk, they can write about it! That’s all I have to say!” – QU29, Child

“Uhm, most kids have grown up with iPads now, if you think about it.” – QU06, Child

Some parents reported that the existing pain scales were useful, and that their children understood them well and that they were used appropriately (3). However, more parents noted that they did not find the scales useful (7), particularly for younger children. Reported issues included a lack of understanding, difficulty with engaging with the scale, and that the pain scales were limited by a child’s pain experience.

“She will always tell me exactly a score for how painful she thinks it is. It’s really good” – QU29, Parent

“Yeah, you didn’t really need to use a scale with me. You can tell she’s in excruciating pain or when it’s like…you know.” – QU01, Parent

“I guess it's hard because if they’ve never really had pain before then what is 10 you know. It’s so subjective even you know like for an adult a man’s probably going to say 10 and a woman would say 2.” – QU35, Parent

Parents’ ideas for pain scales focussed on moving away from numbers to either something children could understand more easily or would enjoy interacting with. It was suggested that using picture scales (7) may improve understanding, with suggestions including images of actual injuries, a whole-body scale (rather than faces), and other ideas such as tactile scales and using recorded voices. Ideas focussed on encouraging engagement were use of colours (9) – either a traffic light system or light to dark colours, using familiar characters on the pain scale – such as Disney characters, Bluey, or animals, or making more interactive scales – including physically moving the scale or using toys. Other creative ideas that emerged were using surrogate scales that the child was familiar with as a more interesting substitute – for example the weather with rainbows as low pain and thunder as painful. Parents also noted that parents should contribute to pain assessment and reported that parental intuition could be as useful as a scale.

Parents reported that technology could be an aid to understanding children’s pain, and could encourage engagement with assessment, particularly for children with special needs (3). However, parents noted a concern regarding technology being a barrier from a useability (1) or financial (1) perspective.

“Yeah, maybe like an app where that can touch the screen where they can touch the colour, where it hurts. Especially for those who are non-verbal. With special needs who can’t express, or have those higher pain thresholds, but its, yeah. I don’t know. But an app would be good.” – QU11, Parent
